# The Causes and Treatment of Abortion

**Published:** 1908-11-28

**Authors:** Thomas G. Stevens

**Affiliations:** Physician to Out-Patients Queen Charlotte's Lying-In Hospital; Physician, Hospital for Women, Soho Square; Obstetric Tutor St. Mary's Hospital, Paddington


### HOSPITAL CLINICS.

### THE CAUSES AND TREATMENT OF ABORTION.

By THOMAS G. STEVENS, M.D., B.S.,Lond., M.R.C.P.Lond., F.R.C.S.Eng., Physician to Out-Patients Queen Charlotte's Lying-In Hospital; Physician, Hospital for Women, Soho Square; Obstetric Tutor St. Mary's Hospital, Paddington.

(A Lecture given at the London Polyclinic, October 21, 1908.)

For all practical purposes the term abortion may be accepted to mean premature expulsion of the ovum at any time before the embryo is viable. In this lecture it is proposed to deal particularly with the causes and treatment of abortion, but the question of diagnosis will have to be considered to some extent. It is a fact that the causes of abortion are sometimes obscure, and in a particular case it may happen that a cause cannot be assigned at all. This probably means that we do not know sufficient about the present state of health of the patient, or some facts in her previous history have escaped her, or our, attention. It is, however, a certainty that abortion does not occur at all easily in an absolutely healthy patient, and a cause can generally be found if a careful search in all directions is made. Women are in the habit of assigning the blame for an abortion to some trivial accident or mental emotion, and although we cannot state that these never are the actual causes, we may accept as an axiom that such accidents are not likely to cause abortion unless there is also present some predisposing cause; in other words, that the individual is not healthy in every respect. It is not possible to draw up a list of predisposing, as opposed to exciting causes, because it is a fact that the lesion which predisposes to abortion may also be the actual cause. An attempt, however, may be made with propriety to classify the causes of abortion, and to indicate their importance and mode of action.

Most abortions will be found to fall naturally into four classes, namely: (1) Those associated with uterine congestion and endometritis; (2) those the result of syphilis; (3) those caused by general diseases; (4) those the result of accidents.

1. Uterine congestion and endometritis is undoubtedly the commonest cause of abortion, and although it occurs most commonly in women who have had children, is certainly to be found in nulliparous women and virgins. Hence a first pregnancy may end in abortion on this account. Along with uterine congestion and endometritis, those lesions of the uterus which cause or accompany these conditions must be reckoned—chronic infection, gonorrhœal or otherwise, retroversion and retroflexion. Fibro-myomata, subinvolution, mucous polypi, salpingo-oophoritis and cervical lacerations, are the common lesions which are associated with endometritis, and these therefore may be reckoned as important causes of abortion. In virgins endometritis may be the sequela of a chronic vaginitis in infancy, following perhaps an exanthem such as scarlatina, measles, or diphtheria, whilst almost any general infection such as enterica or pneumonia may set up a uterine infection, which becomes chronic. Why uterine congestion and endometritis should cause abortion is not at first sight clear, but it is not difficult to formulate a possible reason based on the pathology of these affections. Most important in this respect must be the condition of the capillaries in the endometrium, which in uterine congestion are widely dilated. This must give rise to undue vascularity of the decidua when an embryo becomes emplanted, and as a result the widely dilated thin-walled vessels must ever predispose to hæmorrhage. In fact in this condition any sudden change in the blood pressure, which may be brought about by a trivial accident or emotion, will possibly end in rupture of one of these vessels and consequent hæmorrhage into the chorio-decidual space. Such a hæmorrhage coagulates and acts as a foreign body in the uterus, stimulating it to contract. The compression and flattening of this clot by contractions tends to separate the chorion from the decidua with the result that more hæmorrhage occurs, very soon in sufficient quantity to escape externally. If the chorion is sufficiently detached, death of the embryo follows the hæmorrhage, and expulsion sooner or later must occur. If on the other hand the uterus can be made to tolerate the presence of a blood clot, abortion may be staved off, and the embryo may continue to live and grow. Therefore it follows that hæmorrhage must be the initial stage of abortion in this class and embryonic death must be a secondary occurrence.

2. Syphilis is accepted as a cause of abortion, and perhaps is the most widely known cause of repeated abortion, but it is certainly not nearly such a common cause of abortion in general as is uterine congestion. There is very little authoritative teaching as to why syphilis causes abortion, but considering the few facts at our disposal the real reason may be hinted at. First of all it is commonly agreed that syphilis causes first the death of the embryo, and it is not until the uterus makes an effort to expel the dead embryo that any hæmorrhage occurs. Thus the sequence of events is transposed in this class compared with class 1. Secondly, it is paternal syphilis which is responsible for the majority of syphilitic abortions; in other words, it is syphilis of the embryo and its coverings, not of the mother, which is truly responsible for the premature death of the embryo. Thirdly, abortions due to paternal syphilis occur at later and later dates in succeeding pregnancies; in other words, as the syphilitic virus becomes more attenuated, which we know it tends to do, the lesions which ultimately kill the embryo take longer to develop. The real pathological change underlying all syphilitic lesions, whatever their nature, is an endarteritis, that is to say, a proliferation of the internal coat of the smaller arteries, gradually leading to the obliteration of the lumen of these vessels. If it could be shown that there is obliterative endarteritis in the vessels of the chorionic villi in syphilitic abortions, the case would be proved. Unfortunately this is very difficult of proof, for it is very often difficult to demonstrate even in perfectly definite gummata elsewhere. But it is known that the effects of an endarteritis, namely white infarcts of the placenta, are at least as common in syphilitic abortions as in other conditions, and it is therefore almost reasonable to argue that the true cause of syphilitic abortions is an endarteritis of the fœtal vessels. We must not forget that to prove this we should examine the vessels of the fœtus itself as well as those of the chorionic villi, and we seldom have the opportunity of doing this. It is not out of place here to note that a white infarct of the placenta is a whitish or yellowish mass, produced by the deposition of fibrin from the maternal blood upon chorionic villi, which have been deprived of their blood supply by an obliterative endarteritis.

3. Under the heading of general diseases we have to consider first the acute exanthems, such as typhoid fever, scarlatina, pneumonia, measles, diphtheria, and small-pox. It has been long recognised that these diseases are fertile causes of abortion, and it is commonly believed that the high temperatures which accompany them are causes of death to the fœtus. This has been proved experimentally to be possible by artificially raising the temperature of pregnant animals, with resulting death of the embryos and abortion. It is however more than likely that the toxic blood conditions which accompany these fevers are really the cause of the fœtal death and abortion. It is possible that this toxic condition has an immediate effect upon nerve centres causing uterine contractions. Possibly, too, the fœtus *in utero* actually suffers and dies from these complaints, for the bacteria causing some of them have been found in the fœtal circulation. In addition to the exanthems there are certain other general diseases which certainly cause abortion under some conditions; these are tubercle, heart disease, cirrhosis of the liver, Bright's disease, diabetes mellitus, profound anæmia. Some of these diseases do not inevitably produce abortion, and this is especially the case in tubercle and diabetes; but as a purely general statement it must be accepted that there is a considerable liability to premature expulsion of the embryo in all of them.

4. Abortions are said to be very frequently caused by accidents, often of a very trivial nature, such as tripping over a step, stumbling upstairs, over-reaching, etc., and although the belief is so widespread that these accidents will cause abortion, it is very difficult of proof. Quite on the contrary, it seems much more likely that these trivial circumstances would not be likely to cause abortion as long as the uterus is in a healthy condition. It is highly probable that in many cases in which an accident is said to have caused abortion the uterus is not really healthy, but disposed to abortion from the first. Considering how common it is to find congenital retroversion and flexion of the uterus and endometritis, even in virgins, it is not surprising that many young women should be liable to abortion, which would at once be brought about by some trivial accident. The same may be said of shocks and emotions, which are often said to cause abortion. These states must act upon the uterus by sudden changes in the circulation, and if the uterus is already congested, as in class (1), a small hæmorrhage is readily produced by the rupture of a capillary, and abortion occurs in the end. On the other hand, we must concede that abortion may be directly caused by a serious accident, even though the uterus and pelvic organs are normal. Direct injury is a rare event except in criminal cases, although a fair number of cases are on record where abortion has been caused by kicks or blows in the abdomen. These injuries are more likely to act in the later months when the uterus is large and well above the pelvic brim.

In considering the treatment of abortion it is usual to act in a somewhat different manner according to whether the abortion is threatened, inevitable, or incomplete. Of course, this classification of cases implies an accurate diagnosis of the condition, by no means always an easy matter. Abortion may be said to be threatened in a pregnant woman when she has hæmorrhage from the uterus and pain due to uterine contractions. At the same time, as long as the cervix is closed there is always a chance that expulsion of the embryo is only threatened. If, however, the cervix is opening up, and some part of the embryo, its covering, or even only a blood clot can be felt protruding, then abortion as a rule is inevitable. This, however, like many other things in medicine, is not invariable, for it sometimes happens that the os dilates considerably as a result of uterine contractions and permits of the embryo being felt, and yet under appropriate treatment expulsion of it may be staved off. This is more likely to occur where no hæmorrhage has occurred, and in the middle period of pregnancy rather than the early weeks.

The diagnosis of threatened abortion is sometimes difficult because of the impossibility or difficulty of establishing the fact that the patient is pregnant at all. It must never be forgotten that pain and hæmorrhage are symptoms of other conditions besides abortion, and of none more important or serious than extra-uterine gestation. It is quite astonishing how many ruptured tubal gestations or tubal abortions are at first mistaken for simple uterine abortions, and wrongly treated. Fortunately a fatal result does not often occur, for a large percentage of tubal gestations with hæmatoceles recover if treated in an expectant manner. Therefore it must be clearly laid down that the diagnosis of threatened abortion cannot be made until the presence of a simple uterine pregnancy has been established, after a due consideration of the history of the case combined with a complete bimanual examination. When this has been done the treatment of a threatened abortion is well defined. The patient must be kept absolutely at rest in bed, and steps must be taken to prevent or abolish uterine contractions. The best drug for this purpose is opium given by the mouth, or morphia hypodermically. It need hardly be said, however, that this method of treatment requires supervision, and cannot be adopted in a patient who is not likely to be seen again for a week—for example, a hospital out-patient. The amount of opium or morphia necessary can only be ascertained by observation of the effect produced. If the drug produces a cessation of the pain and contractions, the dose may be lessened and given at longer intervals. In my experience no other drug has given the same good results as opium in these cases; viburnum and hydrastis are quite uncertain in their action. It is possible that the combination of three half-drachm doses of calcium lactate with opium may assist by increasing the coagulability of the blood. This drug should be used only after a careful estimation of the coagulation time, an investigation which is troublesome to carry out in the midst of a busy practice. It is a matter of great difficulty sometimes to know how long to keep a patient in bed after an abortion has been averted; it can only be said that it is better to err on the safe side and insist on a full week's complete rest after all bleeding and pain have ceased. While undergoing this treatment the patient must have a non-stimulating diet, and the bowels should be kept open daily by mild laxatives, combined with enemata if necessary.

If, on examination, the os uteri is found to be opening up and some part of the embryo is actually felt to be coming down through it, abortion is practically inevitable in the early and middle periods of pregnancy, especially if hæmorrhage has occurred. The treatment in this case is essentially different. The object to be attained is complete emptying of the uterus by means of normal uterine contractions, and the means of attaining this differ somewhat at different periods of pregnancy. At all times, however, it must be remembered that the administration of ergot is bad treatment, for although sometimes it produces the desired result, it more often leads to the expulsion of the embryo alone; its coverings, the placenta or part of it, or the decidua being retained *in utero*. The reason for this is that ergot tends to cause tonic contraction of the uterus, and may keep the cervical canal in a condition of spasm so that it will not allow all the uterine contents to be expelled. The exhibition of ergot without any other treatment is without doubt a common cause of incomplete abortion and long continued exhausting hæmorrhages. Ergot therefore should not be given at all in abortion until the uterus is empty; there is no harm in giving it then to keep the uterus well contracted. The simple cases to treat are those occurring in the early weeks of pregnancy, when the cervix uteri is felt on examination to be dilated enough to admit the whole of one finger, and the embryo with its coverings is already detached from its uterine attachments. It is an easy matter then to sweep the finger round inside the uterus and hook out the contents, going over the whole surface to see that nothing is left behind. This little operation, however, must be carried out in an aseptic manner. It is an absolute axiom that neither the finger nor an instrument should be put into the uterus at any time unless the vulva and vagina have been made surgically clean and the finger or instrument is aseptic. To ensure this the vulva must be shaved, or the hair clipped very close with a beard-clipper, and then washed with ether soap followed by an efficient antiseptic. Lysol is one of the best antiseptics for this purpose in the strength of one drachm to a pint. The vagina can be cleaned in the same manner, pouring in the ether soap and then swabbing and rubbing the walls with pledgets of cotton-wool which have been previously sterilised by heat or by prolonged soaking in an antiseptic. Instruments must be boiled immediately before use, and bare fingers must not be used. The bare finger cannot be made aseptic for more than a few minutes by any known method without injury, so that it is best to wear a freshly boiled rubber glove. With these precautions the case will almost certainly run an aseptic course. The anxiety and worry which inevitably follow a septic abortion make it imperative for our own sakes that we should adopt the most stringent precautions. The difficult cases to treat are those in which the os uteri is not sufficiently dilated to completely admit a finger, and yet some part of the embryo can be felt. The treatment of this condition is somewhat different in the early weeks of pregnancy—say up to the tenth—from that adopted from the tenth onward. In the early weeks two methods are open to us which may be used according to circumstances and environment. The first, namely, by vaginal plugging, is simple, easy to carry out, and well suited to the busy man in family practice. The second, immediate anæsthetisation, instrumental dilatation and evacuation of the uterus with the finger and flushing curette, is more adapted to hospital practice, and to the man who is in the habit of performing vaginal operations. The first method can be carried out without anæsthesia. The instruments, etc., required are a Sim's speculum, a vulsellum forceps and a pair of long narrow uterine dressing forceps, and a strip of iodoform gauze three inches wide and three yards long which has been kept soaking in lysol solution. (Dry iodoform gauze is not aseptic.) The vulva and vagina must be cleansed, and the cervix seized with a tenaculum through a speculum, and one end of the gauze packed into the lower uterine segment, and then the rest used to fill up the vagina. If the vagina is not well filled a few pledgets of absorbent wool soaked in lysol solution and squeezed dry may be used to complete the packing. A clean diaper should be placed on the vulva and the case left for about twelve hours, unless some reason arises for interference. The plug acts by causing uterine contractions, and in satisfactory cases the ovum will be found intact on the top of the plug when it is removed. If no contractions occur, the plug may be removed, the vagina recleansed, and a fresh plug inserted. In some cases, however, the finger may be used to complete the evacuation if the ovum has not come completely away.

If the second method is to be used, the instruments required are: a Sim's speculum, a vulsellum forceps, Hegar's dilators, a large blunt flushing curette, uterine dressing forceps, ovum forceps, and a strip of iodoform gauze ribbon. The patient is best placed in the lithotomy position, and, after the usual cleansing, the cervix is drawn down with the vulsellum, and dilated up to the size of the largest Hegar's dilator. Then the ovum may be detached with the finger in all directions and either squeezed out bimanually or removed with the ovum forceps. If it is much broken up the flushing curette may be used to wash out the uterus and draw out any remnants. The operation is finished by plugging the uterus with the iodoform gauze, and using part of it to plug lightly the vagina. The plug is removed at the end of 24 hours, and afterwards no douching or other local treatment is necessary. The use of the curette for this purpose may be criticised, but it must be remembered that it is not recommended except to those who are in the habit of performing uterine operations, and it is not used at all with a dilated uncontracted uterus or after the tenth or twelfth week of pregnancy.

Perhaps the most difficult cases of abortion to treat successfully are those occurring after the tenth week of pregnancy—namely, three, four, or five months' abortions. The reason of the great difficulties sometimes met with in these cases is the inert condition of the uterus so often present. Uterine contractions may occur in these cases only at long intervals and then be weak in character. The result is that hæmorrhage may continue unchecked for many days or even weeks, and there arises a great danger of infection and decomposition of the uterine contents, to say nothing of the bloodless condition to which the patient may be brought. In such cases the use of the vaginal plug may be first recommended, and in them thorough packing of the lower uterine segment may be of great assistance. A longer piece of gauze may be necessary, and if only three-yard lengths are carried the second should be tied to the first so as to make a continuous strip. Sometimes this treatment leads to good contractions and complete evacuation, but even if it does not, as a rule the result is some dilatation of the cervix. It is usually necessary to put two fingers into the uterus to clear out embryos of this age, and anæsthesia is advantageous. These operations are always tedious and difficult, as the embryo is likely to break up during removal. The head is the part which gives most difficulty on account of its size and uncollapsible character. The quickest manœuvre is to puncture it with a pair of sharp-pointed scissors, so as to allow it to be flattened out with the fingers and so hooked through the cervix. The placenta may have to be removed piecemeal, and if hæmorrhage is profuse the uterus should be tightly packed, taking care to fill up the fundus first with gauze.

In these abortions at the middle period of pregnancy, hæmorrhage or sepsis are the signs which demand interference; in their absence or where hæmorrhage is slight the cases do not require energetic treatment at all and may be left to nature.

A large proportion of the cases of abortion met with in practice are "incomplete," and they frequently present problems of great difficulty, especially with regard to diagnosis. The usual history is that hæmorrhage has occurred for many days or weeks, little or more, sometimes amounting to "flooding." If one or more menstrual periods had been missed before this bleeding began, an incomplete abortion is to be presumed. In some cases the embryo or a chorionic sac may have been seen, in which case the diagnosis is certain. It is permissible to treat some of these cases with full doses of ergot on an expectant plan, especially if the uterus appears not to be larger than normal. The assumption in such cases must be that the uterus is empty and that the bleeding is the result of simple congestion following subinvolution. If, however, there is any reason to suppose that any product of conception is retained, ergot is quite useless, as such products are always firmly adherent and no uterine contractions will detach them. In such cases the only treatment of any avail is dilatation of the uterus with Hegar's dilators and thorough curettage, first with a blunt wire curette. If very little tissue is removed by this, and no mass of any size can be felt in the uterus, a sharp curette should be used and the whole lining of the uterus cleared away. Any suspicious tissue removed should be preserved in 10 per cent. formalin in normal saline solution, for section cutting and microscopic diagnosis. In this way malignant growths such as chorion-epithelioma and carcinoma of the body of the uterus will be diagnosed at once and furnish timely indications for further more radical treatment. Curettage is a simple operation in such cases, but is liable to be followed by smart hæmorrhage, which, however, can always be arrested by plugging the uterus. The operation must be aseptic, otherwise endometritis will follow.

It may be suggested that the small operations above described are difficult to carry out in a busy general practice. There is some truth in this, unless forethought is exercised and provision made for emergency operations of this kind. It would be a difficult matter to carry out these operations if everything necessary had to be got ready at a moment's notice; with a little thought and ingenuity, however, things may be so arranged that there is nothing to do but to boil the instruments and put the other things in a bag. Sterile swabs may be kept always ready by cutting up a number of squares of absorbent wool in spare time and keeping them in a glass jar soaking in lysol solution. These swabs may be found useful for other minor surgical emergencies. Iodoform gauze ribbon may also be kept in lysol solution. To carry these swabs and gauze to the patient a large bag made of double layers of face towelling, sewn on three sides, leaving one side open is very useful, and may be kept aseptic by preserving it in the lysol solution and boiling it occasionally if it becomes infected. It is better and saves time to boil the instruments at home, to pour off the water and run a little lysol solution over them and then turn them out direct into the lysol-soaked bag along with the swabs and gauze. Carried in this way, the instruments need not be dried or touched until wanted for use, and being wet with lysol will not rust, a very important fact. When the swabs, gauze and instruments have been placed in the bag it should be rolled up, wrapped up in a square yard of jaconet or thin mackintosh and then placed in the handbag. With swabs and gauze always kept ready in this manner, the time consumed in preparations is limited to that necessary for boiling the instruments.

